# Emerging Role of cAMP/AMPK Signaling

**DOI:** 10.3390/cells11020308

**Published:** 2022-01-17

**Authors:** Muhammad Aslam, Yury Ladilov

**Affiliations:** 1 Experimental Cardiology, Department of Internal Medicine I, Justus Liebig University, Aulweg 129, 35392 Giessen, Germany; muhammad.aslam@physiologie.med.uni-giessen.de; 2 Department of Cardiology, Kerckhoff Clinic GmbH, 61231 Bad Nauheim, Germany; 3 DZHK (German Centre for Cardiovascular Research), Partner Site Rhein-Main, 61231 Bad Nauheim, Germany; 4 Heart Center Brandenburg, Department of Cardiovascular Surgery, University Hospital Brandenburg, Medical School Theodor Fontane, Ladeburger Straße 17, 16321 Bernau bei Berlin, Germany

**Keywords:** adenylyl cyclase, cAMP, PKA, EPAC, AMPK, mitophagy, autophagy

## Abstract

The 5′-Adenosine monophosphate (AMP)-activated protein kinase (AMPK) is a natural energy sensor in mammalian cells that plays a key role in cellular and systemic energy homeostasis. At the cellular level, AMPK supports numerous processes required for energy and redox homeostasis, including mitochondrial biogenesis, autophagy, and glucose and lipid metabolism. Thus, understanding the pathways regulating AMPK activity is crucial for developing strategies to treat metabolic disorders. Mounting evidence suggests the presence of a link between cyclic AMP (cAMP) and AMPK signaling. cAMP signaling is known to be activated in circumstances of physiological and metabolic stress due to the release of stress hormones, such as adrenaline and glucagon, which is followed by activation of membrane-bound adenylyl cyclase and elevation of cellular cAMP. Because the majority of physiological stresses are associated with elevated energy consumption, it is not surprising that activation of cAMP signaling may promote AMPK activity. Aside from the physiological role of the cAMP/AMPK axis, numerous reports have suggested its role in several pathologies, including inflammation, ischemia, diabetes, obesity, and aging. Furthermore, novel reports have provided more mechanistic insight into the regulation of the cAMP/AMPK axis. In particular, the role of distinct cAMP microdomains generated by soluble adenylyl cyclase in regulating basal and induced AMPK activity has recently been demonstrated. In the present review, we discuss current advances in the understanding of the regulation of the cAMP/AMPK axis and its role in cellular homeostasis and explore some translational aspects.

## 1. Current Model of cAMP Signaling

### 1.1. Structure

Cyclic adenosine 3′,5′-monophosphate (cAMP) is a ubiquitous and essential intracellular second messenger molecule involved in a wide range of physiological and pathological processes. cAMP signaling pathways consist of (i) cAMP synthesizing cyclases, (ii) cAMP degrading phosphodiesterases (PDEs), and (iii) cAMP effectors. There are two main sources of cAMP: transmembrane adenylyl cyclases (tmACs), which are localized exclusively at the cellular plasma membrane, and intracellularly localized soluble adenylyl cyclase (sAC).

In mammalian cells, there are 9 genes encoding 9 different tmACs (*adcy1-9*) and one gene encoding sAC (*adcy10*). There are two main differences between tmACs and sAC. First, tmACs are sensitive to G proteins and thus, their activity is regulated by hormones and neurotransmitters. sAC is insensitive to G proteins but can be activated by bicarbonate (HCO_3_^−^), making sAC a unique bicarbonate sensor with enzymatic activity [1]. Second, as mentioned above, tmAC localization is restricted to the plasmalemma, whereas sAC is widely distributed within the cytosol, and has also been found in organelles, e.g., mitochondria and the nucleus [2].

PDEs are key players in cAMP signaling as they are responsible for the termination of the cAMP signal by hydrolyzing the phosphodiester bond to form 5’-AMP. Among 11 mammalian PDE families, three selectively hydrolyze cAMP (PDE4, 7, and 8), and five hydrolyze both cAMP and cGMP (PDE1, 2, 3, 10, and 11) [3].

In eukaryotic cells, there are four known specific downstream cAMP effectors: protein kinase A (PKA), cAMP-dependent exchange protein (EPAC), cyclic nucleotide-gated channels, and Popeye domain-containing proteins [4]. PKA and EPAC are the main cAMP effectors and play essential roles in the regulation of a variety of physiological functions.

The specificity and selectivity of cAMP signaling is maintained by the formation of multiple intracellular cAMP functional compartments within the cell and organelles. This compartmentalization is achieved, first, by distinct spatial distribution of the two main cAMP-generating enzymes, i.e., tmAC and sAC. Under physiological conditions, tmAC produces cAMP predominantly close to the plasma membrane, except in rare cases of tmAC internalization [5]. In contrast, sAC forms cAMP pools within various cellular compartments, e.g., cytosol, mitochondria, nucleus, or in the subplasmalemmal compartment [2,6,7]. Of note, the spatial distribution of sAC is a dynamic process, and under some stresses, the sAC localization is changed [8,9]. Second, the compartmentalization of cAMP signaling within the cell is further supported by the activity of PDEs that restrict cAMP diffusion from its origin [10,11]. Third, scaffolding proteins, such as A-kinase anchoring proteins (AKAPs) play a key role in coupling cAMP synthesis to functional effectors, such as PKA and EPAC. AKAPs are distinguished by their ability to bind PKA. AKAPs also bind other kinases, cyclases, PDEs, G-protein-coupled receptors, and phosphatases (for review, see [4]). Therefore, due to their localization at specific subcellular sites, AKAPs play a critical role in maintaining the subcellular compartmentalization of cAMP signaling.

### 1.2. Regulation

The regulation of cAMP signaling is primarily achieved by activation or inhibition of cyclases. As mentioned above, tmAC activity is controlled by G-proteins, i.e., Gs and Gi [12,13]. In addition, a number of other modulators such as PKA, protein kinase C, and Ca^2+^/calmodulin protein kinase may affect tmAC activity [4,14]. sAC is insensitive to G proteins but can be activated by HCO_3_^−^ [1,15] and the divalent metal cations, Mn^2+^ and Ca^2+^ [15]. Because the sAC function to synthesize cAMP is dependent on the presence of ATP, sAC senses the ATP concentration [16]. When the cellular ATP level is reduced, the activity of sAC is decreased due to substrate limitation [16]. A unique property of sAC is its activation by bicarbonate anions, which suggests its significance in the sensing of metabolic activity. Indeed, human sAC in the presence of 10 mmol/L ATP was stimulated up to 30-fold with a half-maximal effect of ~11 mmol/L bicarbonate, which is appropriate for sensing physiological bicarbonate levels of 2–25 mmol/L [17]. It is noteworthy that sAC activity increases synergistically in the presence of HCO_3_^−^ and Ca^2+^ [17,18]. Thus, increased metabolic activity leading to enhanced bicarbonate formation accompanied by an elevated cellular Ca^2+^ concentration, as occurs in the case of muscle work, will promote sAC activity. The functional significance of sAC activation, however, is still poorly understood, but recent reports argue for key roles of sAC in promoting mitochondrial ATP synthesis [19], apoptosis [20], proliferation [21,22] and hypertrophy [23]. Below, we also describe the role of sAC in regulating AMPK activity.

Another essential mechanism regulating activity of cAMP signaling is its subcellular compartmentalization due to the formation of distinct multiprotein microdomains. As noted above, AKAPs play a key role here. Such multiprotein complexes, also known as signalosomes, include cyclases, PDEs, cAMP effectors (PKA and EPAC) and their targets, allow for precise regulation of target protein activity (for review, see [4,24]).

Finally, spatiotemporal regulation of cAMP signaling is also controlled by PDE activity. A detailed discussion of the regulatory mechanisms controlling PDEs, which is beyond the scope of the present review, can be found elsewhere [25].

## 2. AMPK

5′-Adenosine monophosphate (AMP)-activated protein kinase (AMPK), a highly conserved and adaptive enzyme complex, is a sensor of the cellular energy status that regulates cellular metabolism and energy homeostasis. Mammalian AMPK senses the energy status by monitoring cellular AMP, ADP, and ATP levels [26]. A reduction of cellular ATP content and a corresponding increase in AMP levels (high AMP/ATP ratio) results in an activation of AMPK, which restores the energy balance by inhibiting anabolic processes that consume ATP and promoting catabolic processes that generate ATP [27].

### 2.1. AMPK Structure

**Figure 1:**
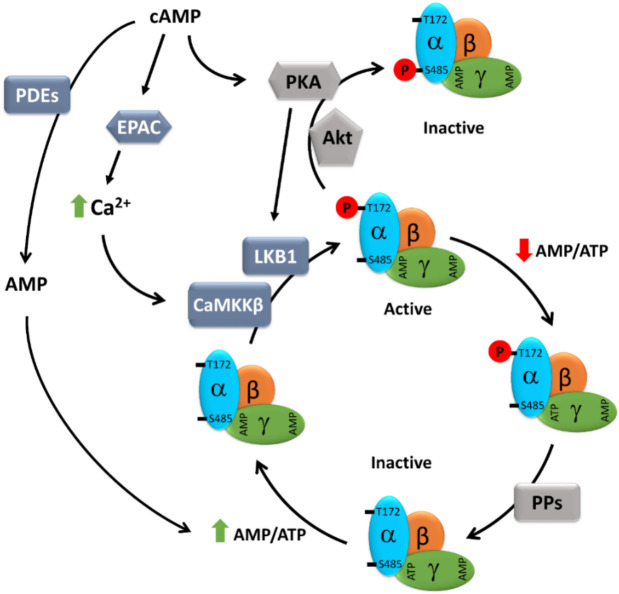
Schematic presentation of the cAMP-dependent regulation of AMPK activity. AMPK consists of three subunits: one catalytic subunit alpha and two regulatory subunits, beta and gamma. At a high AMP/ATP ratio, ATP bound to the γ-subunit is exchanged for AMP, causing an allosteric modification of AMPK that leads to reduced access of Thr172 to phosphatases, but easy access to LKB1 and CaMKKβ, resulting in enhanced AMPK phosphorylation and activation. cAMP, either via EPAC-dependent activation of CaMKK2 or PKA-dependent activation of LKB1, may promote AMPK activity. On the other hand, both PKA and Akt can also directly phosphorylate AMPK at inhibitory Ser485, thus negatively regulating its activity. Furthermore, cAMP elevation may lead to the simultaneous elevation of AMP, a degradation product of cAMP resulting from PDE activity, which, via an increasing AMP/ATP ratio, may promote AMPK activity. AMP: adenosine monophosphate; ATP: adenosine 5’-triphosphate; cAMP: cyclic AMP; CaMKKβ: Ca^2+^/calmodulin regulated kinase kinase beta; EPAC: exchange protein directly activated by cAMP; LKB1: liver kinase b1; PDEs: phosphodiesterases; PKA: protein kinase A; PPs: protein phosphatases.

AMPK is a heterotrimeric enzyme complex consisting of a catalytic α-subunit, a scaffolding β-subunit, and a regulatory γ-subunit (Figure 1). In humans, the α- and β-subunits have two isoforms each (α1, α2 and β1, β2), whereas the γ-subunit has three isoforms (γ1, γ2, and γ3) [28,29]. Combination of different subunit isoforms gives rise to 12 different AMPK isoforms that are differentially expressed and regulated throughout the human body [29,30].

Each α-subunit contains an N-terminal Ser/Thr kinase domain and an autoinhibitory domain that is connected to the C-terminal domain via an adenine nucleotide sensor segment termed the “α-linker” [31,32]. The α-subunit contains a critical residue (Thr174 in the AMPK α1 subunit and Thr172 in the AMPK α2 subunit) that is phosphorylated by an upstream kinase, leading to the enzyme’s activation [31,33,34,35].

The β-subunit contains a carbohydrate-binding module at its C-terminus, allowing AMPK to associate with glycogen bound to glycogen synthase, which is inactivated by AMPK [36,37]. The carbohydrate-binding module also serves as binding site for some pharmacological activators of AMPK, such as A769662 [38]. Moreover, the β-subunit is myristoylated at a conserved MGNXXS myristoylation sequence located at the N-terminus [39] that is involved in the recruitment of AMPK to the mitochondria and regulation of mitophagy [40].

The γ-subunit contains four cystathionine-β-synthase (CBS) tandem repeats that include the binding sites for adenosine-containing regulatory ligands (AMP, ADP, and ATP) [41,42]. Each γ -subunit can bind to three adenosine-containing molecules, thus enabling AMPK to response to changes in the AMP-to-ATP ratio [42] and thus to serve as a direct energy sensor [43].

### 2.2. AMPK Regulation

Binding of AMP to the γ-subunit activates AMPK by three mechanisms: (i) allosteric activation [44,45,46], (ii) enhanced phosphorylation at Thr172 (or Thr174) by upstream kinases (e.g., LKB1) [33], and (iii) reduced dephosphorylation by protein phosphatases [47].

In energy-replete conditions (low AMP/ATP ratio), the CBS domains in the γ-subunit of AMPK are occupied by ATP, exposing the Thr172 of the α-subunit for easy access to phosphatases that keep it in a nonphosphorylated, inactive state. However, under energy-deficient conditions (high AMP/ATP ratio), ATP bound to the γ-subunit is exchanged for AMP, causing an allosteric modification of AMPK that leads to reduced access of Thr172 to phosphatases, but easy access to kinases (mainly LKB1), resulting in enhanced AMPK phosphorylation and activation.

LKB1, an upstream kinase, is constitutively active and the key component of the mechanisms by which AMPK senses the energy status of the cell [33,48]. In addition to energy stress-mediated activation of AMPK via LKB1, AMPK is also phosphorylated and activated by signaling mechanisms that cause a rise in intracellular Ca^2+^ levels. In this case, however, LKB1 does not play a role and AMPK is directly phosphorylated by Ca^2+^/calmodulin-dependent protein kinase kinase 2 (CaMKK2) [34,49,50]. CaMKK2 is activated by a rise in intracellular Ca^2+^ levels that occurs in response to several agonists in specific cell types, such as neurons or endothelial cells [34,49,51].

In addition to activation by phosphorylation at Thr172, inhibitory phosphorylation of AMPK has also been reported. Insulin mediates inactivation of AMPK by AKT-mediated phosphorylation at Ser485 of the AMPK α1-subunit in rats (Ser487 in humans), but not of the AMPK α2-subunit, which blocks upstream kinases from phosphorylating Thr172 [52,53]. Likewise, leptin inhibits AMPK by inducing p70S6K-dependent phosphorylation of AMPK at Ser491 of the α2-subunit [54]. Finally, PKA phosphorylates Ser173 of the α1-subunit of AMPK block upstream kinases, such as LKB1 and CaMKK2, from phosphorylating Thr172, thus negatively regulating AMPK activation [55,56]. A summary of AMPK regulatory mechanisms is presented in Figure 1.

## 3. Regulation of AMPK Activity by cAMP Signaling

An increasing amount of data points to pathways linking cAMP and AMPK. The first evidence suggesting this link emerged from studies in adipocytes. Yin et al. demonstrated that cAMP elevation induced by forskolin promotes AMPK phosphorylation at Thr172 [57]. In agreement, Omar et al. [58] showed in primary adipocytes that isoproterenol-induced cAMP elevation leads to AMPK activation in a PKA- and EPAC-dependent manner [58]. Later, Fu et al. [59] described that treatment of hepatocytes with taurocholate increases cAMP levels, leading to activation of EPAC and its downstream molecules Rap1 and MEK [59]. This signaling pathway has been directly related to LKB1 and AMPK activation. Similar effects were observed with forskolin treatment. Another group investigating the metabolic effects of resveratrol in skeletal muscle cells reported that resveratrol acts as a PDE inhibitor, enhancing cAMP levels and leading to activation of AMPK in an EPAC1-dependent manner [60]. In particular, the authors showed that activation of EPAC1 increases intracellular Ca^2+^ levels and promotes CaMKK2 activity, which phosphorylates AMPK. Similar to this study, Chen et al. [61] demonstrated a role for cAMP in the resveratrol-induced activation of AMPK in endothelial cells. The authors suggest the involvement of PKA here, although the role of EPAC has not been investigated. In agreement, two reports [62,63] argue for a role of PKA-dependent AMPK activation through the LKB1 kinase in follicular thyroid cancer. Altogether, the data suggest positive regulation of AMPK activity by cAMP signaling, particularly via phosphorylation of AMPK at Thr172 in an EPAC- or PKA-dependent manner. Interestingly, under some conditions, PKA may inhibit AMPK by direct phosphorylation at Ser173, which blocks upstream kinases such as LKB1 and CaMKK2 from phosphorylating Thr172 [55].

Although the role of cAMP signaling in the regulation of AMPK activity has been demonstrated in several cell types, little is known about the cAMP compartments controlling AMPK activity. sAC is localized in the cytosol and nucleus, i.e., in close proximity to the AMPK localization sites. In contrast, activation of tmAC by hormones or agonists in healthy cells leads to local, subplasmalemmal cAMP elevation due to the prevention of cAMP diffusion by PDEs. Therefore, co-localization of sAC and AMPK in certain subcellular compartments is consistent with sAC being involved in AMPK regulation by cAMP. In a recent study, we challenged this hypothesis using a cardiac cell line and primary coronary endothelial cells [64]. We found that sAC knockdown significantly reduced basal AMPK phosphorylation and activity in an EPAC- but not a PKA-dependent manner. In agreement, bicarbonate-dependent sAC activation enhanced AMPK phosphorylation, whereas activation of tmAC with forskolin had no effect on the AMPK phosphorylation in cardiac cells. In line with this finding, negative effects of tmAC stimulation on AMPK activity have also been identified in several cell types by other studies that were attributed to the PKA-dependent phosphorylation of the AMPK α-subunit at Ser173, Ser485/491, or Ser497 [55,65,66].

The discrepancy with previous reports demonstrating AMPK activation by tmAC stimulation in adipocytes or hepatocytes [57,58,59] may be due to the differences in cell type, cAMP effectors involved (EPAC or PKA), compartment of cAMP action, or the duration and intensity of cAMP signaling. In particular, excessive cAMP elevation may lead to the simultaneous elevation of AMP, a degradation product of cAMP resulting from PDE activity, which is an important activator of AMPK. Indeed, Chen et al. [67] have shown in mouse oocytes that forskolin-induced cAMP elevation was accompanied by AMP elevation as a result of PDE activity, resulting in AMPK activation.

In summary, numerous reports argue for the existence of a cAMP/AMPK axis in various cell types. cAMP leads to AMPK activation via PKA-LKB1 or EPAC-CaMKKβ axes, yet an inhibitory effect of the direct AMPK phosphorylation by PKA at Ser173, Ser485/491, or Ser497 has been described [55,65,66]. Our recent study further suggested that sAC rather than tmAC is a key source of cAMP for AMPK activation, at least in cardiac cells.

Although cAMP/AMPK signaling is the topic of the present review, one should note here that under some conditions, AMPK may affect cAMP signaling. Particularly, Johanns et al. [68] have demonstrated that in primary mouse hepatocytes, activation of AMPK leads to phosphorylation at several serine residues of PDE4B and its activation followed by reduction in cellular cAMP concentration. Therefore, it is tempting to speculate that this mechanism may serve as a negative feedback loop (i) to prevent sustained or extensive cAMP signaling upregulation or (ii) to protect against AMPK overactivation by cAMP signaling.

## 4. Functional and Translational Significance of the cAMP/AMPK Axis

### 4.1. Mitochondrial Biology

As a metabolic sensor, AMPK contributes markedly to the homeostasis regulation of the main cellular energy supplier, i.e., the mitochondria. AMPK regulates mitochondrial homeostasis at different levels. In particular, AMPK promotes activity and expression of peroxisome proliferator-activated receptor γ coactivator 1 alpha (PGC-1α) [69,70], a key transcriptional factor regulating expression of the nuclear-encoded mitochondrial proteins. Furthermore, AMPK regulates the mitochondrial fusion/fission balance. For example, AMPK promotes mitochondrial fragmentation in response to energy stress via phosphorylation of mitochondrial fission factor at Ser155 and Ser172 [71], a mitochondrial outer-membrane receptor for dynamin-related protein 1 (DRP1). The authors demonstrate that AMPK-dependent phosphorylation of mitochondrial fission factor promotes DRP1 recruitment to the mitochondria, which is required for mitochondrial fission. Mitochondrial fission is an important initial step in mitophagy, i.e., elimination of damaged mitochondria via autophagy. Finally, AMPK promotes autophagy and mitophagy via phosphorylation of Unc-51-like kinase-1 (ULK1), the upstream kinase involved in the formation of the autophagosome. Therefore, AMPK is involved in the whole process of mitochondrial homeostasis, from biogenesis until clearance.

Numerous studies argue for the key role of the cAMP signaling in mitochondrial biology and homeostasis [72,73]. However, only a few reports have linked cAMP signaling to the regulation of mitochondrial homeostasis by AMPK. Park et al. [60] showed that inhibiting PDE4 by resveratrol or rolipram results in activation of EPAC1, increases intracellular Ca^2+^ levels, and activates the CaMKK2-AMPK pathway. As a result, mitochondrial biogenesis and function are improved, which in turn protects against diet-induced obesity and glucose intolerance in mice. In line with these findings, Hamidie et al. [74] have recently shown that intraperitoneal curcumin treatment in rats increased cAMP levels in muscle due to the inhibition of PDE4A. As a consequence, activation of the PKA-LKB1 axis followed by phosphorylation of AMPK and deacetylation of PGC-1α leads to the induction of mitochondrial biogenesis in skeletal muscle.

We have recently demonstrated that intracellularly localized sAC is involved in regulating mitochondrial homeostasis by cAMP/AMPK signaling [64]. In rat cardiac and endothelial cells, we found that sAC knockdown reduced AMPK phosphorylation and activity under basal conditions, which was accompanied by the impairment of mitophagy, mitochondrial depolarization, and mitochondrial reactive oxygen species formation. Activation of EPAC, but not PKA, attenuated the downregulation of phosphorylated AMPK and restored mitochondrial homeostasis. Therefore, the cAMP/AMPK axis markedly supports mitochondrial homeostasis via promotion of mitochondrial biogenesis, function, and clearance. Furthermore, our study [64] provides evidence for the key role of sAC.

### 4.2. Lipid Metabolism

cAMP is a key mediator of metabolic regulation. Caloric restriction or physical activity increases the cellular cAMP concentration, mainly due to the action of glucagon and catecholamines. Other interventions leading to the elevation of cellular cAMP, such as treatment with PDE inhibitors, support fitness and health. Indeed, several reports have shown positive health effects of PDE4 inhibitors in animal models, including improved memory [75] or protection against diet-induced obesity and glucose intolerance [60]. Interestingly, the beneficial effects of cAMP elevation were related to AMPK activation. As a key intracellular energy sensor, AMPK has been implicated as a regulator of lipid metabolism in mammals. In particular, AMPK can modulate lipolysis, lipogenesis, and fatty acid synthesis through phosphorylation of key substrates [76]. A seminal study by Yin et al. [57] linked isoproterenol-induced lipolysis to the cAMP/AMPK axis in adipocytes. Follow-up studies in adipocytes or hepatocytes confirmed this finding and demonstrated involvement of PDEs and PKA in cAMP-induced AMPK activation and lipolysis [58].

The cAMP/AMPK axis also negatively regulates lipogenesis, as has been shown by Ben-Shlomo et al. [77] in hepatocytes treated with glucagon-like peptide-1. Furthermore, cAMP/AMPK signaling promotes fatty acid metabolism by increased expression of genes involved in fatty acid oxidation in hepatocytes [78], although in this study the involvement of glucagon-stimulated extracellular cAMP elevation in AMPK activation was suggested. Wang et al. [79] demonstrated an important translational aspect of the lipid metabolism regulation by cAMP/AMPK axis in mice: the PDE4 inhibitor rolipram increased the cAMP concentration and decreased senescence-associated adipose deposition and inflammation in the liver and kidney that were due to a metabolic disorder. The rolipram-induced increase in cAMP levels in this study also leads to AMPK activation in a CaMKK2-dependent manner. Interestingly, activation of AMPK that induces NAD+ production and enhances activity of sirtuins leading to deacetylation and activation of transcription factors, like PGC-1α [80], is involved in lipid metabolism.

Likewise, in the white adipose tissue of PDE3B knockout mice, an upregulation of cAMP/PKA and AMPK signaling was observed [81]. This was accompanied by reduced weight gain, reduced fat deposits, and improved beta-oxidation in a high-fat diet experimental mouse model, suggesting a direct role of cAMP-mediated AMPK activation [81].

### 4.3. Ischemia

Wan et al. [82] reported that intraperitoneal resveratrol administration significantly reduced the harmful effects of cerebral ischemic injury in rats induced by middle cerebral artery occlusion. Moreover, levels of ATP, phosphorylated AMPK, total SIRT1, and cAMP were increased by resveratrol as well as by a PDE4 inhibitor. The study also suggested a role of cAMP/AMPK/SIRT1 signaling, although the causal role of cAMP in AMPK activation has not been confirmed. Further, in this context, Gao et al. [83] recently showed that C1q/tumor necrosis factor-related protein-3 (CTRP3) treatment protected cultured hippocampal neurons against hypoxia-induced apoptosis via activation of the AMPK/SIRT1/PGC-1a pathway. Activation of cAMP signaling was not investigated in this study, but it has previously been demonstrated that CTRP3 activates cAMP/PKA signaling and protects against oxidative stress-induced brain injury in a rat model of stroke in vivo [84].

The protein hormone adiponectin was found to ameliorate myocardial reperfusion injury via cAMP/PKA signaling [85]. Applying the same model, the authors found that adiponectin also activated AMPK [86]. The authors suggest, however, that adiponectin-induced protection was not AMPK-dependent, because in a mouse model, overexpressing a dominant negative α2-subunit of AMPK, the protection was not abolished [86]. Nevertheless, one may suppose that residual, still-active α1-subunits of AMPK may contribute to the protection.

In a hindlimb ischemia model, treatment with caffeine-activated cAMP/PKA/AMPK signaling, led to enhanced mitochondrial biogenesis (Drp1 activation) and angiogenesis [87]. However, the mechanism of PKA-mediated AMPK activation was not examined in this study. Accordingly, Tseng et al. [88] showed that cilostazol, a PDE3 inhibitor, improved blood flow in a hindlimb ischemia model via cAMP/PKA-dependent activation of AMPK.

In summary, accumulating evidence argues for the existence of cAMP- or AMPK-mediated protection against hypoxia/ischemia-induced cellular injury both in vitro and in vivo. Some studies show a direct link between cAMP and AMPK signaling.

### 4.4. Inflammation

Inflammation is an adaptive response triggered by noxious stimuli and conditions such as infection and tissue injury [89]. Activation of an acute inflammatory response is a fundamental requirement to eradicate the threat; however, uncontrolled chronic inflammation is integral to the pathogenesis of a variety of chronic disease processes, including cardiovascular diseases. For example, low-grade inflammation of the vascular wall is associated with endothelial dysfunction and plays a key role in the pathogenesis of atherosclerosis [90,91]. In recent decades, research has focused on understanding the mediators and mechanisms of chronic inflammation. cAMP is known as a mediator of anti-inflammatory responses, and cAMP-dependent signaling has been pharmacologically exploited for the treatment of inflammatory diseases [92]. Moreover, emerging data demonstrate the anti-inflammatory role of AMPK signaling [93,94].

NF-κB is a key transcription factor whose activation promotes the expression of pro-inflammatory genes [95]. It has been shown that signaling mechanisms raising cellular cAMP levels can abrogate NF-κB activation via both cAMP/PKA and cAMP/EPAC pathways [96,97,98,99]. Likewise, AMPK signaling also modulates NF-κB signaling in various cell types [93]. However, few studies have directly investigated the role played by the cAMP/AMPK axis in modulating NF-κB signaling. For example, Chen and colleagues investigated the adiponectin-mediated inhibition of NF-κB signaling in endothelial cells and showed that adiponectin activates cAMP/PKA signaling, which leads downstream to an activation of AMPK and inhibition of NF-κB signaling [100].

MicroRNAs (miRs) are also involved in inflammatory responses. In a mouse model of lipopolysaccharide-induced acute lung injury, Hu et al. [101] showed an upregulation of miR351-5p that was accompanied by suppression of cAMP/PKA and AMPK signaling. Administration of an antagomir to miR351-5p abrogated this lipopolysaccharide-induced suppression of cAMP/PKA and AMPK signaling, resolved inflammation, and improved mouse survival [101]. However, the mechanism of the miR351-5p-mediated suppression of cAMP signaling was not investigated, although the mRNAs of adenylyl cyclase 1 (Adcy1), Adcy5, and Adcy6 contain seed binding sites for miR351-5p in 3′-UTR regions [101], indicating potential downregulation of tmAC expression.

### 4.5. Type 2 Diabetes

Glucagon-like peptide-1 (GLP-1) mimetics and GLP-1 receptor (GLP-1r) agonists are widely used drugs for glycemic control in type 2 diabetes [102]. GLP-1 mimetics sensitize pancreatic beta cells for insulin secretion in response to physiological stimuli. The mechanism is in part dependent on activation of cAMP signaling. The GLP-1r is also expressed in heart and vasculature, and its activation is involved in anti-inflammatory and cardiovascular protective signaling [103,104,105]. The main pathways activated by GLP-1r signaling include cAMP and AMPK [106]. Although cAMP/PKA- and cAMP/EPAC-mediated AMPK activation in GLP-1/GLP-1r signaling has been proposed [107,108], there is a lack of experimental evidence showing direct interaction of GLP-1r-induced cAMP signaling with AMPK signaling, and this needs to be explored.

Ma et al. [109] investigated the protective effects of CTRP3 in streptozotocin-induced diabetic cardiomyopathy in rats. In addition, employing a H9C2 cell culture model, the authors demonstrated that CTRP3-activated cAMP/EPAC signaling led to an activation of AMPK signaling downstream of LKB1 activation. The authors proposed that CTRP3-mediated protection against diabetes-induced cardiomyopathy is via cAMP/Epac-dependent AMPK activation.

In a mouse model of metabolic abnormalities and diabetes (KK-Ay mice), Xu et al. [110] demonstrated that coenzyme Q10 supplementation inhibited weight gain and improved lipid metabolism and insulin resistance [110]. These protective effects were accompanied by the suppression of liver PDE4 expression and upregulation of cAMP content as well as AMPK phosphorylation. The authors suggested that these effects were mediated via cAMP-mediated activation of AMPK and reduction in oxidative stress. However, the causal role of cAMP/AMPK signaling was not investigated in the study. In a further study, exposure of cultured human retinal endothelial cells to high-glucose concentrations resulted in reduction in phosphorylated AMPK levels that were normalized by the EPAC agonist, suggesting an EPAC-dependent activation of AMPK [111].

In contrast to the above-described studies, in cultured mouse primary hepatocytes, an activation of cAMP/PKA signaling leads to suppression of AMPK activity via enhanced phosphorylation at inhibitory S485 of the AMPKα1 subunit [112]. This results in an enhanced glucose production by hepatocytes and a suppressed metformin-mediated anti-hyperglycemic effect. This study suggests that both cAMP and AMPK signaling play a balancing role in maintaining the stable level of hepatic glucose production.

### 4.6. Miscellaneous

Pain: In a rat pain model, chronic compression (paw) injury resulted in miR142-3p-induced downregulation of AC9 with corresponding reduction in tissue cAMP as well as in phosphorylated AMPK levels [113]. Treatment with forskolin (a direct activator of AC) partly rescued the reduction in AMPK phosphorylation and ameliorated sciatic nerve inflammation.

Reproduction: In boar spermatozoa, elevation of cAMP levels resulted in an upregulation of AMPK phosphorylation that was abrogated by pharmacological inhibition of PKA [114], suggesting a role of cAMP/PKA signaling in AMPK activity and sperm motility.

Senescence: Cell senescence is a hallmark of aging. SIRT1 is generally accepted to be an antisenescence enzyme that elicits its effects by the deacetylation of a wide range of target proteins that control various cellular functions [115,116,117]. SIRT1 expression/activity is upregulated by cAMP and AMPK signaling [118,119,120,121]. Alternatively, pharmacological SIRT1 activators may promote activity of cAMP and AMPK signaling [122,123]. Recently, Sung et al. [124] demonstrated that pharmacological activation of SIRT1-reduced cell senescence in aortic smooth muscle cells via induction of AMPK phosphorylation at its inhibitory site Ser485 was dependent on cAMP/PKA signaling [124]. The phosphorylation of AMPK at its activating site Thr172 or AMPK activity was not measured in the study; therefore, it cannot be concluded whether there was any change in AMPK activity. PKA-mediated AMPK phosphorylation at Ser485 and corresponding reduction in phosphorylation at Thr172 and activity of AMPK have previously been demonstrated in INS-1 cells and mouse embryonic fibroblasts [65].

## 5. Conclusions and Perspectives

AMPK plays a key role in cellular energy homeostasis under basal conditions as well as during stress and in disease states. During the past decade, emerging data have demonstrated the regulation of AMPK by cAMP signaling. We have learned that the cAMP/AMPK axis is involved in the regulation of a number of key cellular processes. This pathway controls mitochondrial biogenesis as well as metabolic activity. Moreover, by modulating cellular metabolic activity, it may prime the organs and tissues for protection against ischemic injury and for the early resolution of inflammation. Several pharmacological agents, natural as well as synthetic, which activate cAMP signaling (e.g., resveratrol, GLP1, and CTRP3) also induce activation of AMPK signaling that is, at least in part, dependent on cAMP signaling. In this scenario, the cAMP/AMPK axis offers an important druggable target for various diseases.

Physical activity may be the alternative, “drug-free” approach to promote the cAMP/AMPK axis. Indeed, physical exercise leads to elevation of cellular cAMP levels via activation of either tmACs, as a result of hormonal stimulation of Gs-coupled receptors, or sAC, as a result of cellular Ca^2+^ and HCO_3_^−^ elevation. Our recent report [64] demonstrated the existence of a bicarbonate-sAC-AMPK axis and its contribution to mitochondrial homeostasis. Thus, promotion of this axis, either by physical activity or by hypercapnia, may be a useful approach for treating some diseases that are accompanied by mitochondrial dysfunction.

## Data Availability

Data are available on request.
